# The kinome of pineapple: catalog and insights into functions in crassulacean acid metabolism plants

**DOI:** 10.1186/s12870-018-1389-z

**Published:** 2018-09-18

**Authors:** Kaikai Zhu, Hui Liu, Xinlu Chen, Qunkang Cheng, Zong-Ming (Max) Cheng

**Affiliations:** 1 College of Horticulture, Nanjing Agricultural University, Nanjing, 210095 Jiangsu China; 2 Department of Plant Sciences, University of Tennessee, Knoxville, TN 37996 USA; 3 Department of Botany and Plant Pathology, Central Oregon Agricultural Research Center, Oregon State University, Madras, OR 97741 USA

**Keywords:** Alternative splicing, Coexpression network, Crassulacean acid metabolism, Duplication events, Expression patterns, Phylogenetic relationship, Pineapple kinases

## Abstract

**Background:**

Crassulacean acid metabolism (CAM) plants use water 20–80% more efficiently by shifting stomata opening and primary CO_2_ uptake and fixation to the nighttime. Protein kinases (PKs) play pivotal roles in this biological process. However, few PKs have been functionally analyzed precisely due to their abundance and potential functional redundancy (caused by numerous gene duplications).

**Results:**

In this study, we systematically identified a total of 758 predicted PK genes in the genome of a CAM plant, pineapple (*Ananas comosus*). The pineapple kinome was classified into 20 groups and 116 families based on the kinase domain sequences. The RLK was the largest group, containing 480 members, and over half of them were predicted to locate at the plasma membrane. Both segmental and tandem duplications make important contributions to the expansion of pineapple kinome based on the synteny analysis. *Ka/Ks* ratios showed all of the duplication events were under purifying selection. The global expression analysis revealed that pineapple PKs exhibit different tissue-specific and diurnal expression patterns. Forty PK genes in a cluster performed higher expression levels in green leaf tip than in white leaf base, and fourteen of them had strong differential expression patterns between the photosynthetic green leaf tip and the non-photosynthetic white leaf base tissues.

**Conclusions:**

Our findings provide insights into the evolution and biological function of pineapple PKs and a foundation for further functional analysis of PKs in CAM plants. The gene duplication, expression, and coexpression analysis helped us to rapidly identify the key candidates in pineapple kinome, which may play roles in the carbon fixation process in pineapple and help engineering CAM pathway into C3 crops for improved drought tolerance.

**Electronic supplementary material:**

The online version of this article (10.1186/s12870-018-1389-z) contains supplementary material, which is available to authorized users.

## Background

CAM plants such as pineapple, agave, and kalanchoe can inhabit water limited areas by reducing transpirational water loss in day-time and improve water use efficiency (WUE) [1]. These plants shift part or all carbon dioxide (CO_2_) fixation to the dark period catalyzed by phosphoenolpyruvate carboxylase (PEPC) during stomata opening [2, 3]. Control of nocturnal stomata opening and carbon fixation can help CAM plants to have higher WUE than C3 even C4 plants to adapt drought environment. The CAM plants use just one-sixth water consumed by C3 plants, and a quarter of water consumed by C4 plants [1]. To meet the food demands of a greater global population with the rising temperatures that may lead to more dry areas and crop production loss, improving crop WUE becomes a more important strategy for coping with drought. Therefore, understanding the CAM photosynthesis may help to fully elucidate CAM mechanism and help to engineering CAM strategy into crop plants in environment adaption and crop production. CAM plants are distributed in over 400 genera of plants, and all the enzymes in the CAM pathway can be found in C3 plants, suggesting that CAM evolved from a C3 ancestor [1]. However, the detailed divergence after emerging from C3 ancestor is still not clear [1].

One of the protein groups that play key roles in CAM is protein kinases (PKs) that regulate activities of downstream target proteins via phosphorylation [4]. The PKs commonly possess a conserved catalytic domain, which consists of 250 to 300 amino acid residues [5]. Hanks and Hunter [6] first functionally classified the eukaryotic PKs based on the phylogeny analysis of catalysis domains. Lehti-Shiu and Shiu [7] classified the plant kinase superfamily, plant kinome, from 25 plant species into various groups based on their kinase domain sequence comparison. The plant kinomes are commonly greater than animal kinomes [7]. More than 1000, 1500, and 2000 kinases were identified in *Arabidopsis*, rice, and soybean, respectively [8–10], comparing to only about 500 PKs in the human genome [11]. The RLK/Pelle is the largest group in land plant kinomes [12], for example, over 600 in the *Arabidopsis* kinome [8, 13]. However, only four Interleukin Receptor-Associated Kinases (IRAKs) exist in the human kinome, which are most closely related to plant RLK/Pelle [12]. Extensive expansion of RLK/Pelle group was considered to make the major contribution to the large size of plant kinome [7]. The expansions of plant PK families were likely due to recent duplications and divergence events, including whole genome, segmental and tandem duplications [7, 10, 14].

Phosphorylation is a common post-translational modification for regulating enzyme activity, protein stability and protein-protein interactions [15]. PKs have been documented to play essential roles in regulating plant development, metabolic processes, cell cycle, and responses to biotic and abiotic stresses [7, 16]. Phosphoenolpyruvate carboxylase kinase (PPCK), which regulates the phosphoenolpyruvate carboxylase (PEPC) activity in a circadian manner by phosphorylation, plays a central role in primary CO_2_ fixation in CAM plants [1, 3]. However, the study of phosphorylation process related to the carbon fixation in CAM pathway is still largely lacking [1].

With the released whole-genome sequences and extensive transcriptome data of pineapple (*Ananas comosus*), an important tropical perennial CAM monocot fruit crop [3, 17], it allows us to fully characterize the entire pineapple PKs, a CAM plant kinome, and their tissue-specific and temporal expression patterns. A precise annotation of plant PK genes is the first step toward fully understanding their roles in plant development and environmental stress responses [18–20].

In this study, we identified entire pineapple PKs and classified into groups and families based on their kinase domain sequences. The sequence features and expansion mechanisms were also analyzed. In addition, the tissue-specific and diurnal expression patterns of pineapple PKs were evaluated with their coexpression relationships. Our results provide a global view of pineapple kinome and a foundation for further systematic characterization of CAM pathways in this important tropical fruit crop.

## Results

### Genome-wide identification and classification of protein kinases in pineapple

**Fig. 1:**
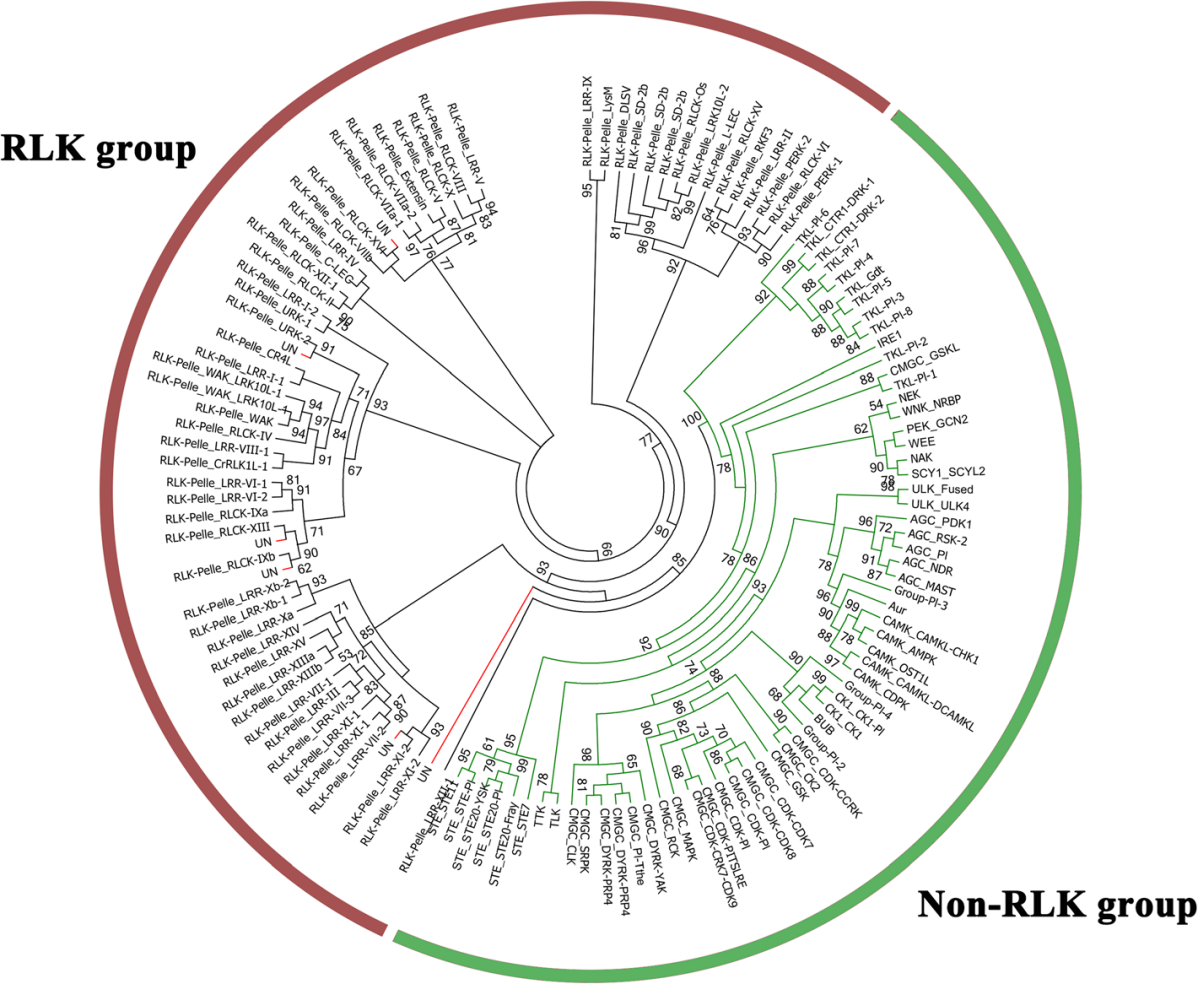
Classification and phylogenetic analysis of pineapple PK families. The maximum-likelihood tree was constructed by amino acid sequences of the kinase domain using the FastTree. Branches were colored to represent two different groups. The non-RLK groups are marked with green branch; the RLK group is marked with red branch. The detailed phylogeny was provided in Additional file 2: Figure S1

A total of 758 pineapple kinase proteins were identified (Additional file 1: Table S1) after the redundant sequences were excluded. The 758 PKs were further classified into groups and families by HMM search approach, and seven out of the 758 genes showed a different result with the HMM search results (Additional file 2: Figure S1) after examined by the phylogenetic analysis. These PK genes were not clustered with any of other known families and thus placed in an unclassified group (Additional file 1: Table S2). The remaining 751 PKs were divided into 20 groups and 116 families (Additional file 1: Table S3, Fig. 1). The RLK members (480) represented more than half of the total PKs, and constituted the largest group in pineapple kinome, which could be further classified into 55 families. The other six major groups included AGC (23), CAMK (57), CK1 (15), CMGC (67), STE (28), and TKL (48). Among the 116 kinase families, 33 families just contained 1 member, and the RLK-Pelle_DLSV family was the largest family, which contained 41 members.

### Intron numbers, chromosomal locations, conserved domains and subcellular localizations of pineapple PKs

After classifying the 758 PK genes into families, related gene structures of different family members were determined to gain insights into the structural diversity of pineapple PK genes. Intron numbers of pineapple PK genes (Additional file 1: Table S2) varied widely, from 0 to 67. *Aco006620* (RLK-Pelle_DLSV) contained the most introns. Most PK genes contained at least one intron, only 72 intronless genes were found in pineapple kinome, and 156 PK genes contained more than 10 introns (Additional file 1: Table S2). In the family level, members in RLK-Pelle_LRR-VII-1, RLK-Pelle_LRR-Xb-1, RLK-Pelle_RLCK-X, RLK-Pelle_RLCK-XIII, RLK-Pelle_URK-1, and TKL-Pl-7 contained the same numbers of introns. However, the intron numbers in some other families were highly variable. For example, twelve genes in CAMK_CAMKL-CHK1 family contained less than three introns per gene, whereas each of the remaining seven family members contained 12 to 15 introns per gene. The phylogenetic tree also revealed that the CAMK_CAMKL-CHK1 family could be also divided into two clusters in alien with the intron numbers (Additional file 2: Figure S1), intron-rich cluster (> 8 introns per gene) and intron-less cluster (< 3 introns per gene), suggested that the exon/intron distribution patterns seemed to relate to the evolution of this family (Additional file 1: Table S2).

Among the 758 pineapple PK genes, except that 43 genes were still in scaffolds, the remaining 715 PK genes were mapped to all 25 pineapple chromosomes (Additional file 3: Figure S2). The chromosome location distribution appeared to be uneven. Chromosome 5 contained 55 pineapple PK genes, followed by 53 on chromosome 1 while chromosome 24 just encompassed 4 PK genes. The other chromosomes all encompassed more than 15 PK genes.

The subcellular location information of a gene product might be used to predict the functions. Since the subcellular localizations of the pineapple PK genes were still largely unknown, we predicted the subcellular localization of the PKs with CELLO and LOCALIZER (Additional file 1: Table S2). Based on the result with CELLO, about 38% (287/758) PKs predicted to localize to the plasma membrane, and more than half of the RLK members (277/480) were predicted to the plasma membrane (Additional file 3: Figure S3). About 70% members in AGC and STE group were localized to the nucleus. 67% CAMK group members were predictably localized to the cytoplasm and 53% CK1 group members were localized to the mitochondria, respectively. A large part of CMGC and TKL group members were localized to the nucleus or cytoplasm. The subcellular localization result with LOCALIZER summarized that 43.1% PKs were located in the nucleus without transit peptides, 51 and 69 were localized in the chloroplasts and mitochondria, respectively (Additional file 1: Table S2). In the non-RLK group, 60.4% of PKs were localized to the nucleus. However, 48.1% of genes in the RLK group were localized to the nucleus.

Conserved domains in pineapple PKs were further detected against the Pfam database. Totally 377 PKs just had one kinase catalytic domain. The remaining PKs with additional conserved domains were detected in the AGC (82.61%), CAMK (68.42%), RLK (57.92%) and TKL (56.25%) groups, indicating that various families contained multiple domain compositions (Additional file 1: Table S4). Members in each family generally shared similar conserved domain arrangements, suggesting common evolutionary history within the same family. Interestingly, nearly all PKs that contained Pkinase_Tyr (PF07714) domain occurred in RLK and TKL groups.

Among the 70 pineapple PKs that contained multiple kinase domains (Additional file 1: Table S5), 57, 9, and 3 members contained 2, 3, and 4 kinase domains, respectively. Aco014466 encompassed the most kinase domains (5 kinase domains). Pineapple PKs that contained more than one kinase domain concentrated in several families. For example, all four members in CMGC_SRPK family and 80% (12/15) AGC_RSK-2 members contained two kinase domains.

### Segmental and tandem duplication events among pineapple kinome

**Fig. 2:**
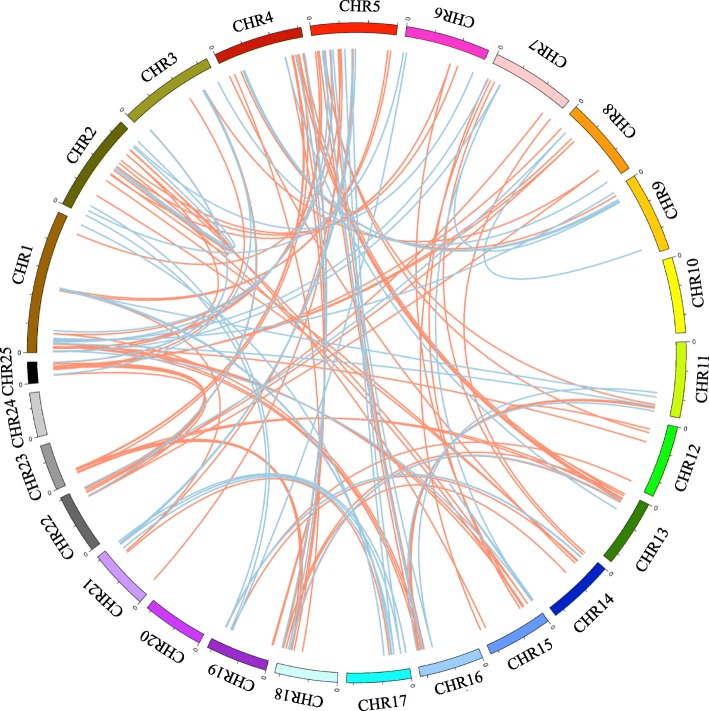
Segmental duplications of pineapple PK genes. Twenty-five pineapple chromosomes were displayed in different colors, and the chromosome number was indicated at the top of each chromosome. Segmentally duplicated genes were linked by two different colored lines. Red lines suggested all segmental duplication events in RLK group, and the blue lines indicated segmental duplication events in non-RLK group in pineapple kinome

**Fig. 3:**
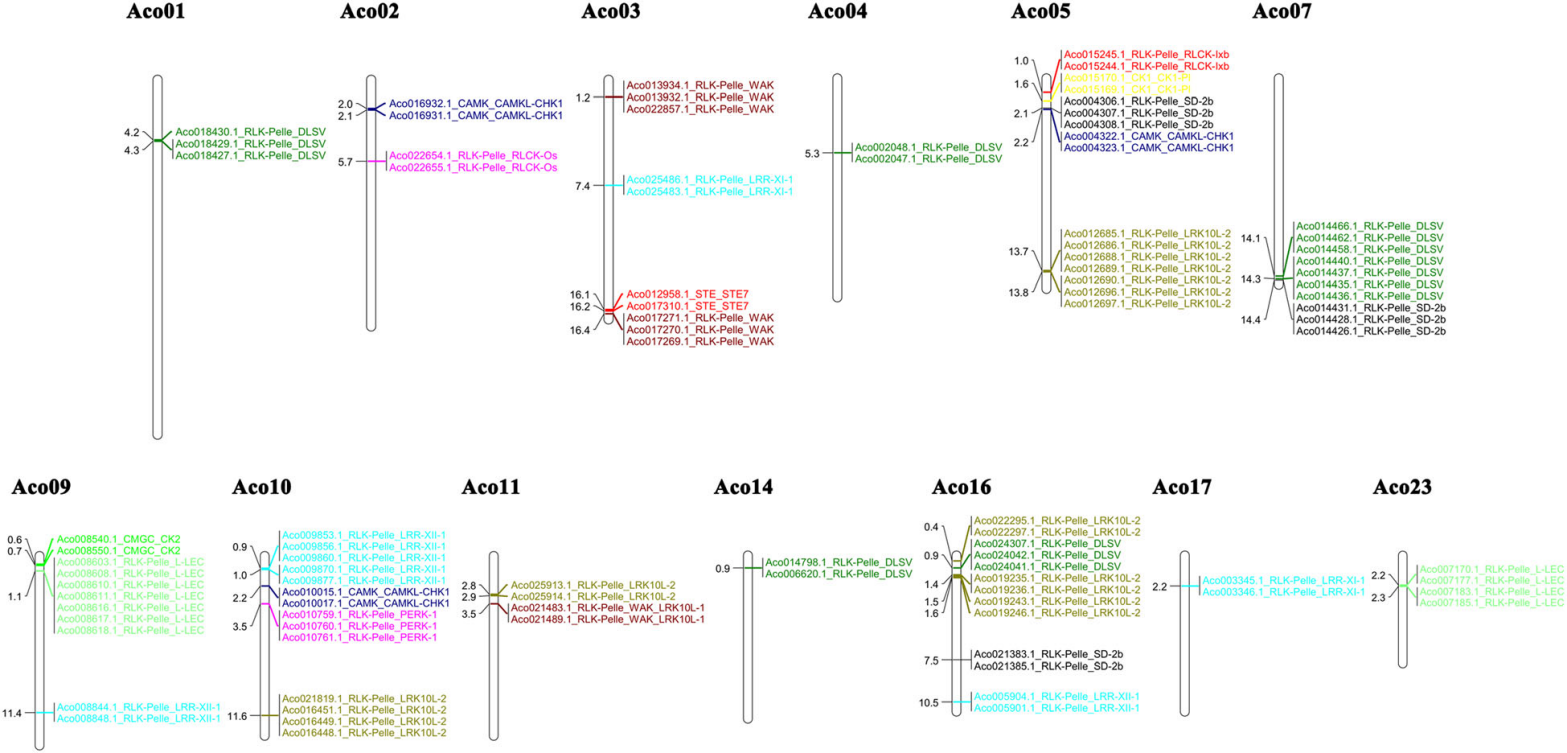
Chromosomal locations of the 95 tandemly duplicated PK genes in pineapple. They were distributed unevenly among the 13 pineapple chromosomes. Gene IDs with corresponding family names were indicated to the right of each chromosome, and related information on gene location is listed on the left

Gene duplication plays a central role in the expansion of a large kinase superfamily, and contributed to help plants to get novel functions, such as adaptation to the environmental stress [8]. The pineapple kinome had 135 segmental duplication events with 228 PKs (Fig. 2, Additional file 1: Table S6), with 85 occurred in the RLK group. Seventy tandem duplication events were identified with 95 PK genes on the 13 chromosomes (Fig. 3, Additional file 1: Table S7). The number of tandemly duplicated PK genes in each chromosome varied from 2 to 16, with chromosome 5 containing the most tandemly arrayed PK genes.

Since genes from tandem duplication might function in stress response, and in contrast to other duplication types, tandem duplication occurred more frequently [21]. Gene ontology (GO) functions of the 95 tandemly duplicated genes were further analyzed. Three main GO categories are biological processes, cellular components, and molecular functions. Surprisingly, all the tandemly duplicated genes were involved in two of the three GO categories, biological processes and molecular functions (Additional file 3: Figure S4A). Functional GO terms for the 95 tandemly duplicated PK genes were further analyzed (Additional file 3: Figure S4B). The top three GO terms included ATP binding (29%), protein kinase activity (27%), and protein phosphorylation (27%). Our results suggested that these tandemly duplicated genes might function in plant development and signal transduction.

**Fig. 4:**
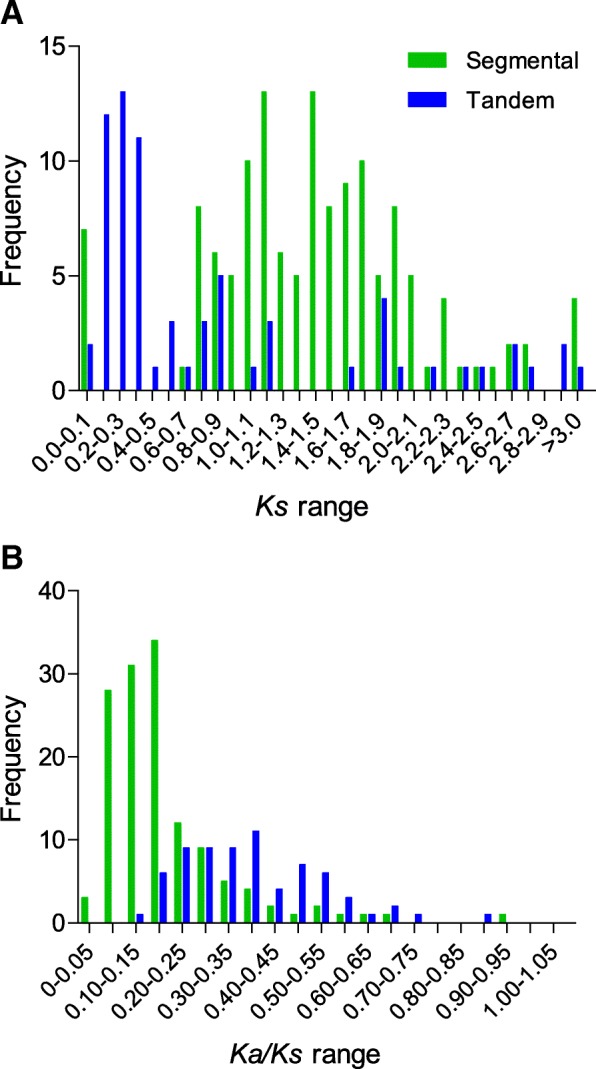
The distribution of relative *Ks* and *Ka/Ks* ratios frequency between segmental and tandem duplication events in pineapple kinome. The X-axis denoted average *Ks* (**a**) or average *Ka/Ks* (**b**) per unit of 0.1; and Y-axis denoted frequency

*Ks* value is the time indicator for duplication blocks, and the frequency distribution of *Ks* values is used to estimate the relative date of genome duplication (Fig. 4a, Additional file 1: Table S8). Among the pineapple segmental duplication events, the *Ks* values peaked at the range from 1.1 to 1.2 and 1.4 to 1.5. However, among the 70 tandem duplication events, *Ks* values peaked at 0.2 to 0.3, showed most segmental duplication events were more ancient than most tandem duplication events. The *Ka*/*Ks* ratio was an effective measure to determine the selection of duplication events. *Ka*/*Ks* = 1, neutral selection; *Ka*/*Ks* < 1 means negative selection, also known as purifying selection; and the value of *Ka*/*Ks* higher than 1 indicates positive selection (Darwinian selection). Interestingly, all the *Ka*/*Ks* values of segmental and tandem duplication events were less than 1, indicating that negative (purifying) selection was the primary influence on the expansion of pineapple PK genes (Fig. 4b).

### Alternative splicing of the pineapple PKs

**Fig. 5:**
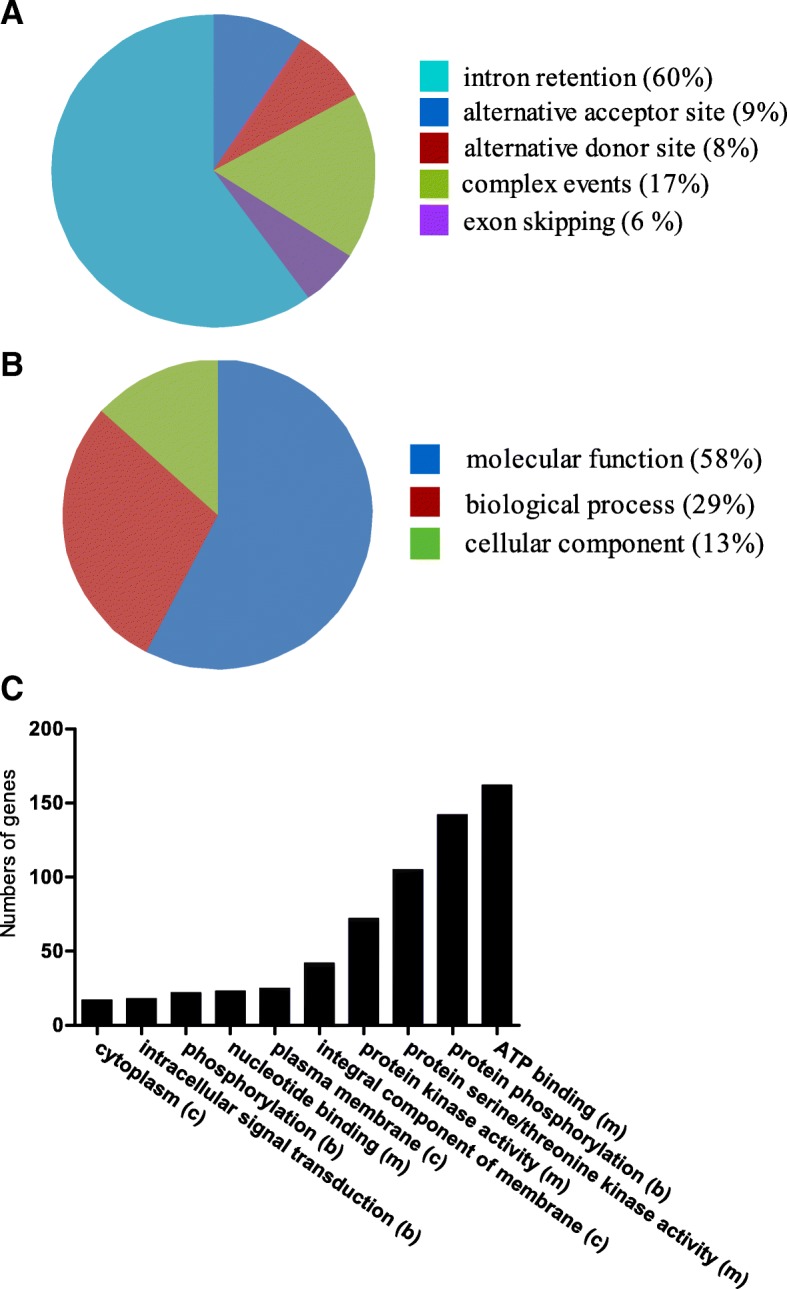
Alternative splicing types of 200 AS genes in pineapple kinome and their GO functional analysis. (**a**) The size of each slice in the pie chart indicated the relative abundance of AS types of AS genes in the pineapple kinome. (**b**) The size of each slice in the pie chart indicated the relative abundance of GO functions of 200 AS genes in the pineapple kinome and their detail classifications (**c**)

Besides gene duplication, alternative splicing (AS) also plays various roles in biological functions, including plant stress adaptation. Analysis of AS genes in pineapple kinome helped us to understand the phosphorylation regulatory mechanisms. Among the 758 PK genes identified in pineapple genome, 200 genes contained alternatively spliced transcripts (Additional file 1: Table S9). Among the different AS events, intron retention accounted for 59.9%. The other three AS types including alternative acceptor site, alternative donor site and exon skipping accounted for 9.2, 7.7 and 5.9%, respectively. The remaining 17.3% were detected as complex events (Fig. 5a).

GO functional analysis on the AS genes (Fig. 5b and c) showed that the largest fractions (58%) of the GO terms were associated with molecular function such as ATP binding, protein serine/threonine kinase activity, and nucleotide binding. The second largest fraction was related to biological processes (29%) including protein phosphorylation, phosphorylation and intracellular signal transduction. Unlike the tandem duplication genes that are only involved in two categories (molecular functions and biological processes), the remaining PK genes that underwent AS (13%) were involved in cellular components category such as integral component of membrane, plasma membrane, and cytoplasm (Fig. 5c).

### Expression analysis of pineapple PK genes during development

**Fig. 6:**
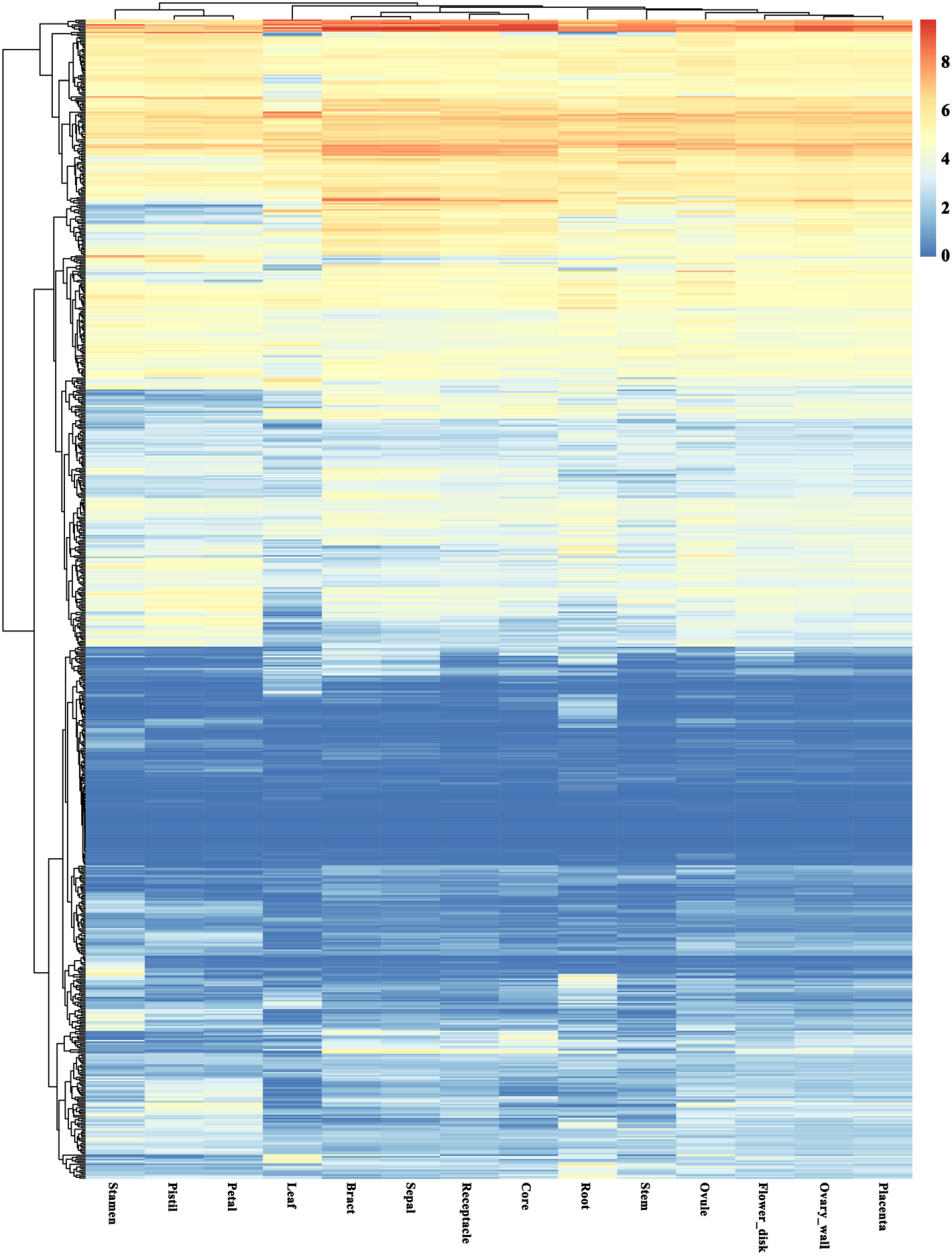
Heatmaps of the expression profiles of pineapple PK genes in 14 different tissues with hierarchical clustering. The heatmaps were generated using R

**Fig. 7:**
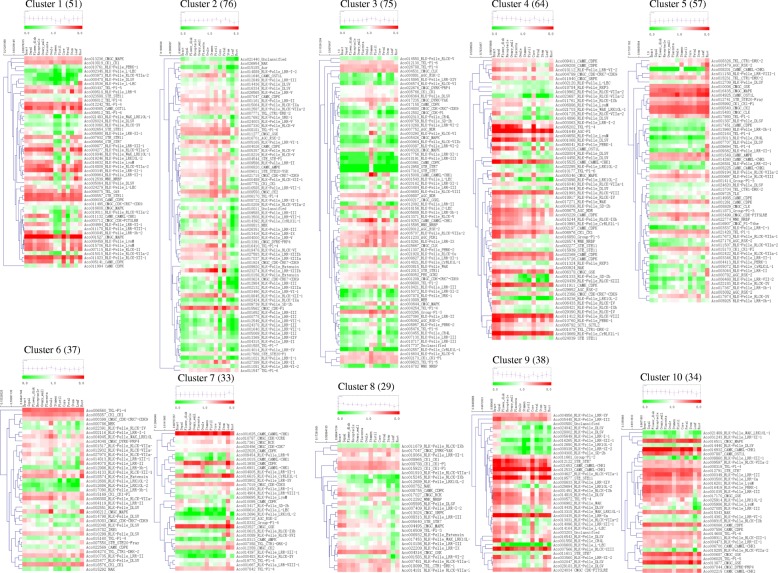
Heatmaps of the 10 clusters generated by clustering the expression patterns of 494 PK genes in different tissues during development. The number at the top of each cluster was the number of PK genes in each cluster. The mean expression pattern of each cluster was performed with a small graph

Tissue-specific expression of different genes could use for functional validation. To understand the expression patterns of PK genes in different pineapple tissues, publicly available transcriptome dataset was analyzed. The expression data of all 758 PK genes in 14 different tissues (Fig. 6, Additional file 1: Table S10) showed that *Aco001649* (AGC-Pl), *Aco001625* and *Aco012533* in CAMK_CAMKL-CHK1, *Aco001527* in CMGC_MAPK, *Aco003435* in RLK-Pelle_LRR-Xa were highly expressed nearly in all detected tissues. In contrast, others, such as *Aco009019* (BUB), *Aco015017* (CAMK_AMPK), *Aco000324* (CK1_CK1), *Aco004806* (RLK-Pelle_CR4L), showed very low expression in tissues examined. The PK genes with low expression in most tissues were found in many different families, especially in the RLK group. Some other genes presented tissue-specific expression patterns. For example, *Aco008201* in CAMK_CAMKL-CHK1 and *Aco006575* in TKL_CTR1-DRK-2 exhibited high expression in root. *Aco000718* in AGC_RSK-2, *Aco010615* in RLK-Pelle_RLCK-IXb, *Aco013938* in CAMK_CDPK, and *Aco010015* in CAMK_CAMKL-CHK1 had a relatively higher expression level in leaf than in other tissues. For analyzing the expression patterns of pineapple PKs among different tissues, and finding the genes which played roles in photosynthesis, we filtered the lowly expressed genes and classified the remaining 494 genes into ten clusters using the *k*-means algorithm with Pearson’s correlation distance based on their expression data (Fig. 7, Additional file 1: Table S11). Most (65.38%) PK genes were grouped into five largest clusters (cluster 1 to 5). PK genes in cluster 7 expressed higher in the leaf tissue than in other tissues, indicated they might function in pineapple photosynthesis.

To compare the different expression patterns of pineapple PK genes in the family level, a heatmap with hierarchical clustering analysis was created using the kinase family expression data (Additional file 3: Figure S5). The pineapple PK gene families showed distinct expression patterns. Several families like Group-Pl-4 and RLK-Pelle_XVI expressed highly in leaf tissue, however, AGC_RSK showed high expression in root. The AGC-Pl, CMGC_GSK, CMGC_CK2, and RLK-Pelle_LRR-Xa families expressed highly in most tissues. However, most families in the RLK group showed low expression levels in different tissues, indicated that although RLK is the largest group in pineapple kinome, few RLK members involved in pineapple development. Tissue coexpression network of the PK families was further constructed (Additional file 3: Figure S6). The tissue network containing 93 nodes and 362 edges was separated into two main and four subnetworks. The RLK-Pelle_RLCK-V contained 33 edges, ranking the top family that had most edges. This result indicated that this family might play a central role in plant development.

### Diurnal expression patterns and coexpression analysis of pineapple PK genes

**Fig. 8:**
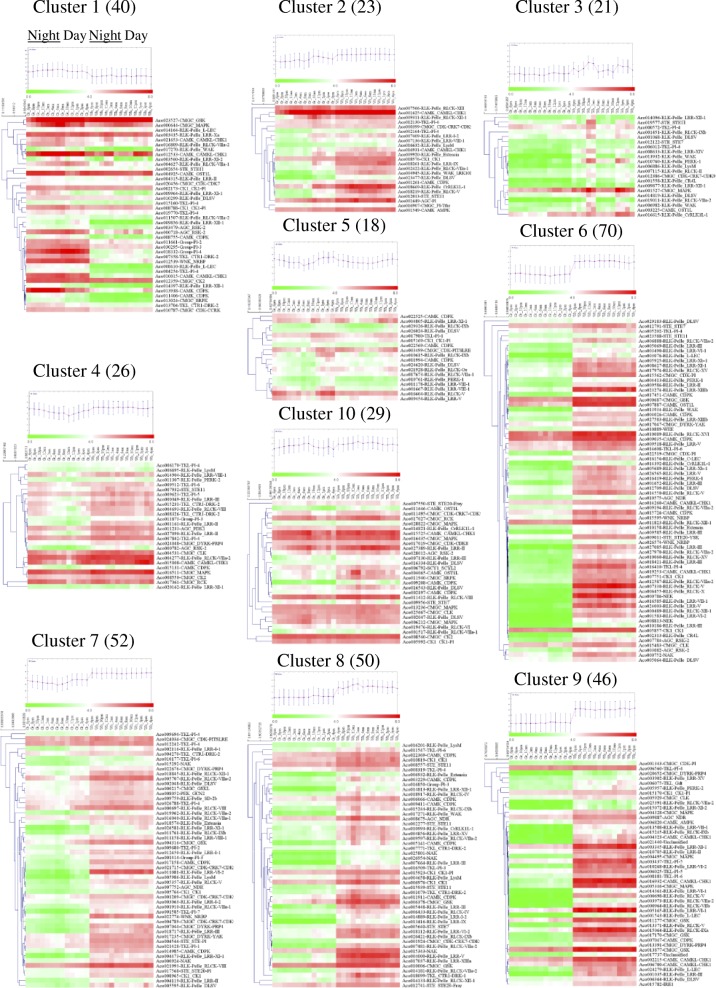
Heatmaps of the 10 clusters generated by clustering the diurnal expression patterns of PKs in green and white leaf tissues. The number at the top of each cluster was the number of PK genes in each cluster. The mean expression pattern of each cluster was performed with a small graph

**Fig. 9:**
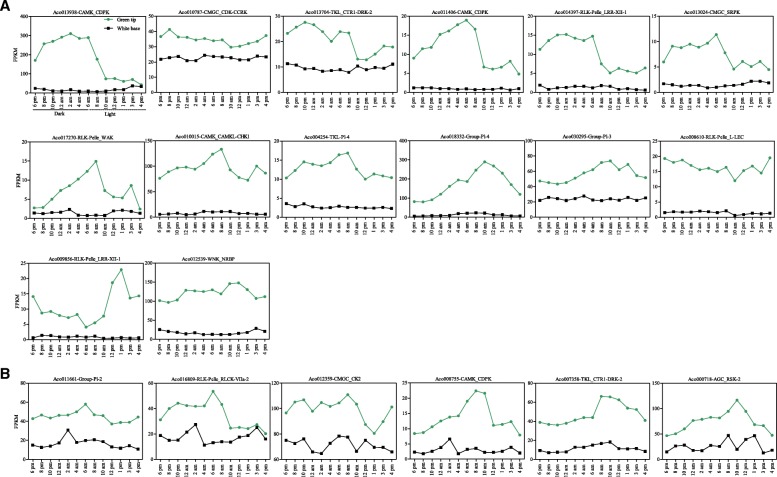
Expression patterns of 20 pineapple PK genes in cluster 1 in both green leaf tip and white leaf base during the 24-h period. The X-axis indicated different time points, and Y-axis indicated FPKM values. (**a**) Genes showed different expression patterns in green tip and white base leaf tissues. (**b**) Genes showed similar expression patterns in the two tissues

Previous studies reported that the circadian rhythms of genes in a CAM plant played an important role [3]. To investigate the diel expression patterns of pineapple PK genes, we specifically analyzed RNA-Seq data of pineapple photosynthetic green tip (Additional file 1: Table S12) and non-photosynthetic white base leaf tissue (Additional file 1: Table S13) at 2-h intervals over entire 24-h period to identify the temporal expression patterns of PK genes. By comparing the expression patterns in the two kinds of leaf tissues, candidate PKs involved in CAM specific carbon fixation process could be distinguished from non-CAM related members that function in other processes, which had a diurnal expression in the photosynthetic tissue with low expression in the non-photosynthetic tissue [3]. After the low expression PK genes were filtered [22], the remaining 375 genes were classified into clusters using the *k*-means algorithm with Pearson’s correlation distance based on their diurnal expression patterns (Fig. 8, Additional file 1: Table S14). Clusters were showed as heatmaps with clustering analysis and the mean expression pattern. Ten different clusters of co-expression patterns across a 24-h period were performed and gene numbers in different clusters varied from 18 (cluster 5) to 70 (cluster 6). Interestingly, genes in cluster one showed higher expression level in green tip (photosynthetic) than in white base (non-photosynthetic) tissue, indicating that genes in this cluster might function in pineapple photosynthetic process (Fig. 8). A co-expression network was further constructed between genes in cluster one that contained 33 nodes with 382 edges (Additional file 3: Figure S7). Each node harbored different number of regulatory edges varied from one (*Aco009964* in RLK-Pelle_LRR-XI-1 and *Aco023527* in CMGC_GSK) to twenty-two (*Aco008755* in CAMK_CDPK). Twenty nodes contained more than 10 edges, suggested that they were tightly correlated. The 20 PK genes were then retrieved and their detail expression patterns performed with FPKM were listed in Fig. 9. Interestingly, all the 20 genes showed at least one peak in green tip tissue during temporal expression. However, 14 genes had different expression patterns between green tip and white base tissues (Fig. 9a), and only 6 genes showed similar expression patterns in white base tissue to that in green tip tissue (Fig. 9b). The 14 PK genes diurnally expressed in photosynthesis part of pineapple leaf were considered to participate in the photosynthesis process. Among the 14 genes, 6 genes, *Aco013938* (CAMK_CDPK), *Aco011406* (CAMK_CDPK), *Aco013704* (TKL_CTR1-DRK-2), *Aco014397* (RLK-Pelle_LRR-XII-1), *Aco010787* (CMGC_CDK-CCRK) and *Aco013024* (CMGC_SRPK), peaked in nocturnal or early morning (Fig. 9a), indicating that they may function in specific CAM photosynthesis pathway and the CO_2_ fixation process at night. The *Aco013938*, also known as a *PPCK* gene in pineapple [3], activated PEPC by phosphorylation during nighttime in pineapple, and this gene also highly expressed in leaf (Fig. 7), therefore, likely participating in CAM carbon fixation pathway [3]. Interestingly, another six genes including *Aco010787, Aco010015* (CAMK_CAMKL-CHK1)*, Aco018332* (Group-Pl-4)*, Aco008610* (RLK-Pelle_L-LEC)*, Aco009856* (RLK-Pelle_LRR-XII-1) and *Aco012539* (WNK_NRBP) also expressed higher in leaf than in other tissues (Fig. 7, Additional file 1: Table S10), indicated that they might be involved in pineapple photosynthetic process. Another surprise was that more than half of the 14 genes in Fig. 9a were generated by segmental or tandem duplication. For example, *Aco013938, Aco011406, Aco013024, Aco010015* and *Aco004254* (TKL-Pl-4) were all generated by segmental duplications. However, four genes including *Aco017270* (RLK-Pelle_WAK)*, Aco018332, Aco008610* and *Aco009856* were derived from tandem duplications (Additional file 1: Table S7)*.* The different expression of duplicated genes in pineapple kinome, might be the result of the neo-functionalization or sub-functionalization [10].

The expression patterns of PK genes which were generated by both tandem and segmental duplications were retrieved after filtering the lowly expressed genes (Additional file 1: Table S15), the coexpression networks based on diurnal expression data of 137 segmentally and 22 tandemly duplicated genes were analyzed (Additional file 3: Figure S8). The coexpression network which was generated by the segmentally duplicated genes (Additional file 3: Figure S8A) contained 106 nodes and 1683 edges, most of the nodes were tightly correlated. 73 nodes contained more than 10 edges, surprisingly, six of them contained over 60 edges, including Aco000489 (RLK-Pelle_RLCK-XII-1, 61), Aco005640 (STE_STE7, 62), Aco003500 (RLK-Pelle_LRR-Xa, 63), Aco008813 (NEK, 63), Aco014181 (RLK-Pelle_RLCK-VIIa-2, 66), Aco015343 (NAK, 66). However, only 13 nodes with 21 edges existed in the coexpression network which were based on the tandemly duplicated genes (Additional file 3: Figure S8B).

## Discussion

### Pineapple kinome possesses a large RLK group

Many cellular processes are controlled by posttranslational modification of specific proteins, and reversible phosphorylation is one of the most widespread posttranslational modifications, performed by various kinases, and controls most signaling pathways [16]. Although some discoveries have been made in elucidating the functions of PKs, a very limited number of kinases have been well demonstrated in CAM plants, probably because the genome sequences of several CAM plants have just been published recently, including *Phalaenopsis equestris* [23], pineapple [3] and kalanchoe [24]. Therefore, genome-wide identification and characterization of PKs help to understand not only the regulatory networks controlling the biological processes, but also the evolutionary driving force leading to biodiversity.

In previous research, PKs were found to represent 1.7% of the genes in the human genome but approximately 4% in plant genomes [11, 25]. Lehti-Shiu and Shiu [7] identified and classified all PKs from 25 plant species, the kinase numbers varied from 326 (*Volvox carteri*) to 2535 (*Eucalyptus grandis*). In this study, 758 pineapple PKs were identified (Additional file 1: Table S1), representing 2.8% (758/27024) in pineapple genome [3]. This proportion is lower than that in *Arabidopsis* (3.4%), grapevine (3.7%) and soybean (4.7%) genome [10, 13, 26]. Similar to 119, 123, 121 and 122 families in *Arabidopsis*, rice, grapevine and soybean kinome, respectively, pineapple kinases were classified into 116 families (Additional file 1: Table S3, Fig. 1) [7, 10, 26]. Three families including AGC_PKA-PKG, CAMK_CAMKL-LKB and SCY1_SCYL1 were absence in pineapple. Among the 116 families, 33 just contained 1 member. Most of these families were also highly conserved in other plants, and they might be involved in more basic cellular processes. For example, expression of *Arabidopsis PEK_GCN2* gene in gcn2 mutant yeast cell can complement amino acid starvation response [17]. IRE1 plays a key role in responding to endoplasmic reticulum stress in mammals, and *Arabidopsis* IRE1 spliced the mRNA of bZIP60 to synthesize the active form of protein [27]. RLK was the largest group in pineapple kinome, accounting for 63.3% PK genes in pineapple (Additional file 1: Table S3). This percentage of RLK in pineapple kinome was similar with that in *Arabidopsis* (60%) [13] and soybean kinome (65.5%) [10]. Since only 2 and 3 RLK members existed in two algae species (*Chlamydomonas reinhardtii* and *Volvox carteri*), respectively, significant expansion of RLK group must have taken place after land plants emerged [7]. Subgroups including RLK-Pelle_LRR and RLK-Pelle_RLCK were the two large subgroups in RLK, which contained 166 and 117 PKs, respectively.

The subcellular localization information showed that more than half RLK members were membrane-located, most likely functioning in response to various extracellular signals (Additional file 1: Table S2). The RLK-Pelle_LRR members had been proved to involve in signaling transduction, immunity, and stress response [28]. However, the RLK-Pelle_RLCK members lacked the extracellular domain, most of them were predicted to localize in the nucleus, mitochondria and cytoplasm (Additional file 1: Table S2), and they were possibly related to plant growth and development [29, 30].

### Expansion and duplication of pineapple kinome

Compared with other eukaryotes, plants usually contain higher rates of gene duplication [21]. Gene duplication contributed largely to the size of plant kinome, especially the RLK group [12]. Most sequenced angiosperm genomes had at least one whole genome duplication (WGD) event during their evolution; tandem and segmental duplications also appeared commonly [28]. The existence of duplicated gene pairs can foster new functions for the expanded genes. Segmental duplication has proved to make the major contribution to the expansion, and accounted for the generation of 30.1% (228/758) in pineapple kinome, especially in the RLK group (Fig. 2). However, 71.4% and 75.0% PK genes were generated by segmental duplication in soybean and *Arabidopsis*, respectively [10, 13].

Tandem duplicates occurred much more frequently compared with WGD, and were important for adaptive evolution to quickly changing environments [21]. Ninety-five tandem duplicated genes were identified, accounting for 12.5% in pineapple kinome, 83 being RLK members (Fig. 3). This percentage is higher than that in *Arabidopsis* (9.5%) and soybean (10.6%) kinome, but lower than that in maize (17.2%) kinome [10, 13, 14]. Gene ontology functional analysis revealed the predicted functions of the tandemly duplicated PK genes, and all the GO terms were related to molecular function and biological processes (Additional file 3: Figure S4). The GO categorization of tandemly duplicated genes in soybean kinome was closely related to biotic/abiotic stress responses and development [10]. Most of the recent expansion of the *Arabidopsis* RLK genes associated with defense/resistance responses was tandemly duplicated genes [25]. Rice and *Arabidopsis* genes in GO categories related to response to abiotic stress tend to be tandemly duplicated [21, 31].

The *Ks* values among segmental and tandem duplication events showed tandem duplication occurred more recently than segmental duplication events, indicating that the tandemly duplicated genes may involve in response to various external stress signals (Fig. 4). The reason for the connection between stress response and tandem duplications is that tandem duplications accepted rapid changes in gene content over a few generations [21].

Alternative splicing, which enables a single gene to generate multiple mRNA products, is a central mechanism for regulating proteome diversity for environmental adaptation [32]. Splice site selection has presented to be involved in cell type, development stage and abiotic/biotic stress [33]. Two hundred PK genes contained AS events accounting for 24% in the kinome (Additional file 1: Table S9), and about 30% AS genes in pineapple genome [22]. The intron retention was the most prevalent AS type in pineapple kinome (Fig. 5), accounting for 60%. Intron retention also accounted for 62% in pineapple genome. In other plant species, intron retention also remains as the main type among the classified AS events [34, 35].

### Expression patterns of pineapple PK genes

Genes with similar expression patterns and functions are commonly co-regulated [36]. Genes that have tissue-specific expression patterns often play central roles during plant development. Tissue-specific RNA-Seq analysis of pineapple PK genes showed various expression patterns (Figs. 6 and 7). The PKs, such as highly expressed CK2, CDPK, MAPK gene families have been proven to function in plant growth and development [37–39]. *Arabidopsis* and rice SnRK1 have regulatory functions in plant growth and development throughout the life cycle [40]. Overexpression of *GhMPK7* in tobacco and *Arabidopsis* indicated *GhMPK7* might be involved in phytohormone-regulated development [19]. Two somatic embryogenesis receptor-like kinase (SERK) genes in pineapple (*AcSERK1* and *AcSERK2*) could be used to monitor the acquisition of embryogenic competence, both of them could be induced by different hormones and abiotic stresses. We retrieved the cds sequences from NCBI with the accession number HM236375 (*AcSERK1*) and HM236376 (*AcSERK2*), respectively [41, 42]. Finally, we identified that *Aco001161* (RLK-Pelle_LRR-II) was *AcSERK1*, and *Aco009586* (RLK-Pelle_LRR-II) was *AcSERK2* using BLASTN against pineapple genome database.

During the daytime, CAM plants keep the stomata closed to reduce the water loss through evapotranspiration, and fix carbon dioxide by PEPC when stomata are open nocturnally [3]. These features of CAM plants increase water use efficiency, and enable them to adapt drought environments. Previous reports have shown that CAM was evolved from a C3 ancestor, indicating that CAM-engineering into C3 is a viable strategy to improve water use efficiency in most C3 crops [1]. Key to the success of this approach is deeply dependent on the understanding of the genomic, biochemical, and physiological characteristics of CAM plants [1]. Kusakina and Dodd [43] indicated that phosphorylation participates in the circadian regulation of plant photosynthesis and plays a key role in plant circadian system. The pineapple PK genes temporal expression pattern in green leaf tip (photosynthetic) and in white leaf base (non-photosynthetic) helped to differentiate the PK roles in pineapple and to select candidate genes involved carbon fixation in CAM plants (Fig. 8). Finally, 20 PK genes were selected and their expression patterns were analyzed (Fig. 9). Among the 20 genes, 14 showed different expression patterns in two leaf parts, and these PK genes were found diurnal expression in green tip tissue with low expression in white base tissue, and were considered to be involved in carbon fixation process in CAM photosynthetic pathway (Fig. 9a). Among the 14 genes, *Aco013938* has been identified as the *PPCK* gene, which is a key gene in CAM photosynthesis pathway and mediate the phosphorylation of PEPC [1, 3]. The *PPCK1* in *Kalanchoë fedtschenkoi,* another CAM plant, was also peaked in the middle of the dark in leaf tissue [24]. The transgenic RNAi line of *KfPPCK1* reduced total CO_2_ fixation in the dark period [44]. The ortholog gene of *Aco011406* (CAMK_CDPK) in *Arabidopsis, AtCPK7,* played a critical role in regulation of water uptake from soil [45]. An ortholog of *Aco018332* (Group-Pl-4), *AtSTN7* (*AT1G68830*), plays an important role in plant response to environmental changes [46]. Surprisingly, *At5g58140*, the ortholog of *Aco000718* in AGC_RSK-2 (Fig. 9b), also known as *PHOT2*, is a blue light photoreceptor in *Arabidopsis* that regulates stomatal opening [24]. Similarly, the ortholog of *Aco016809* (RLK-Pelle_RLCK-VIIa-2), *AT2G28930* is also required for stomatal opening in the light in *Arabidopsis* [47], indicating that *Aco000718* and *Aco016809* may play roles in regulation of stomatal opening in pineapple (Additional file 1: Table S16).

## Conclusion

Totally 758 pineapple PKs were identified and further classified into 20 groups, and 116 families. Duplication events contributed to the large expansion of pineapple kinome. The *Ka/Ks* ratios indicated the duplication events were all under purifying selection. The *Ks* values of segmental duplication events were greater than that of tandem duplication events, demonstrating that tandem duplications occurred more recently. In addition, pineapple PK genes showed various expression patterns in different tissues as well as between day and night in different leaf tissues. Reversible phosphorylation regulating CAM activities is still largely unknown in CAM plants. In this work, we characterized kinome at the global level in pineapple. Our results provided a foundation to investigate the functions of pineapple PKs and could be used to select candidates for engineering drought tolerance in C3 crops.

## Methods

### Computational retrieval and identification of pineapple PKs

For identifying all protein kinases from pineapple genome, all pineapple protein-coding genes were downloaded from phytozome V12.1 (https://phytozome.jgi.doe.gov/). Hidden Markov models (HMMs) profiles of the two Pkinase clan including Pkinase (PF00069) and Pkinase_Tyr (PF07714) downloaded from Pfam (http://pfam.xfam.org/) [48] were applied to investigate putative PKs using HMMER v3.1b2 [49]. The default parameters were adopted with an E-value cut-off of < 1.0E-5, and the potential sequences were further examined by SMART (http://smart.embl-heidelberg.de/) [50]. In this study, a putative PK was considered as a PK if the related kinase domain alignment covered at least 50% of the Pfam domain model [7]. PK classification into groups and families was defined using HMMs of the different families developed by Lehti-Shiu and Shiu [7] who built from four plant model species including *Chlamydomonas reinhardtii*, *Physcomitrella patens*, *Oryza sativa*, and *Arabidopsis thaliana*.

### Sequence alignment and phylogenetic analysis

A phylogenetic tree was built using the kinase domain sequences to confirm the classification result. The kinase domain sequences of all identified pineapple PKs were retrieved using a perl script. Multiple sequence alignment of the kinase domain sequences was performed using the MUSCLE program by MEGA 6.06 [51]. The maximum-likelihood (ML) method was applied to construct the phylogenetic tree of pineapple kinase proteins using FastTree version 2.1.9 (www.microbesonline.org/fasttree/) with default parameters [52].

### Chromosomal locations and intron numbers

The chromosomal locations of pineapple PK genes were retrieved from the pineapple database (https://phytozome.jgi.doe.gov/). Intron numbers of all of the pineapple PK genes were obtained from the gff file from the genome resources.

### Subcellular localization prediction

Protein subcellular localization was predicted by CELLO (http://cello.life.nctu.edu.tw) [53] and LOCALIZER (http://localizer.csiro.au) [54].

### Identification of tandem and segmental duplication events in pineapple kinome

Multiple Collinearity Scan toolkit (MCScanX) package (http://chibba.pgml.uga.edu/mcscan2/) was applied to identify the collinear blocks of pineapple PKs followed on the manual [55]. The segmental duplication events were visualized by Circos 0.69 software (http://circos.ca/). Tandem duplications were defined as at least two genes in a family appearing in neighboring intergenic regions separated by five or fewer genes less than 100 kb [56]. The chromosomal distribution of the tandemly duplicated genes in pineapple kinome was illustrated using Mapchart software (http://www.wur.nl/en/show/Mapchart-2.30.htm).

### GO functional classification analysis of PK genes in pineapple

The Blast2GO tool (https://www.blast2go.com/) was used to obtain the gene ontology (GO) term IDs for pineapple PKs. The annotations of the GO-term IDs were retrieved from the Gene Ontology Consortium (http://www.geneontology.org).

### Calculation of the *Ka*/*Ks* values

The full-length coding sequences of segmentally and tandemly duplicated PK genes were first aligned by ClustalW 2.0 [57]. Then the non-synonymous substitutions (*Ka*) and synonymous substitutions per site (*Ks*) were analyzed using MEGA6.06. The ratio of *Ka* to *Ks* (*Ka/Ks*) was used to determine the selection pressure among duplication events.

### Alternative splicing analysis

The alternative splicing (AS) data of PKs in pineapple genome were collected from Plant Alternative Splicing Database (http://proteomics.ysu.edu/altsplice/), and the AS event types were also analyzed [22].

### Expression analysis

The transcriptome data from Pineapple Gene Database (http://118.24.17.128/html/Pineapple_Expression_DB_By_HeLab_AT_SCAU/) were collected to analyze the expression patterns of pineapple PK genes [58]. The expression data of identified pineapple PKs in 14 different tissues and organs during development were retrieved (NCBI accession number PRJNA382449) [59]. The diurnal expression data over a 24-h period retrieved from two pineapple leaf tissues including green leaf tip (photosynthetic) and white leaf base (non-photosynthetic) with the accession number PRJNA305042 [3]. The expression values of identified pineapple PK genes in different tissues and time points were performed using fragments per kilobase of exon model per million fragments mapped (FPKM) values. Heatmaps representing the log_2_ (FPKM + 1) of PKs from the RNA-Seq data were constructed with hierarchical clustering analysis using the R package (www.r-project.org). To analyze the tissue-specific expression pattern at the family level, gene expression data of all members in each different family were averaged.

To cluster the tissue-specific or diurnal expression patterns of PK genes, the FPKM values of genes less than 10 in all tissues or time points in two different pineapple leaf tissues were considered as lowly expressed genes and filtered [22]. Finally, the remaining PK genes were classified into different clusters by Multiple Experiment Viewer (MEV) version 4.9 software (http://mev.tm4.org/) with *k*-means clustering algorithms [60, 61].

### Coexpression network construction

Pearson correlation coefficient (PCC) values based on various tissues and time course expression data were calculated using IBM SPSS Statistics v24 (https://www.ibm.com/us-en/marketplace/spss-statistics). All the pineapple gene or family pairs whose absolute value of PCC was higher than 0.8 were extracted at the 0.01 significance level (*P*-value) and used for a regulatory network analysis. The coexpression network was performed using Cytoscape v3.3.0 (http://www.cytoscape.org).

## Additional files

Additional file 1: **Table S1.** Kinase domain annotation of 758 pineapple protein kinases. **Table S2.** Family classification of pineapple protein kinases and their related information. **Table S3.** The numbers of pineapple PK genes in different families. **Table S4.** Domain organization of 758 pineapple PKs. **Table S5.** List of 70 pineapple protein kinases containing multiple kinase domains. **Table S6.** Pineapple PK genes generated by segmental duplication. **Table S7.** Pineapple PK genes generated by tandem duplication. **Table S8.** Segmental and tandem duplication events and *Ka/Ks* values of pineapple protein kinases. **Table S9.** Alternative splicing types of PK genes in pineapple. **Table S10.** Average FPKM expression values of 758 pineapple kinase genes in 14 different tissues among developmental stages. **Table S11.** 494 genes in ten clusters with different expression patterns during development. **Table S12.** Average FPKM expression values of 758 pineapple kinase genes during 24-h in green leaf tip. **Table S13.** Average FPKM expression values of 758 pineapple kinase genes during 24-h in white leaf base. **Table S14.** Genes in ten clusters with different expression patterns. **Table S15.** Expression values of pineapple kinase genes generated by duplication during 24-h in both green and white leaf tissues. **Table S16.** The orthologs of 20 pineapple PKs in *Arabidopsis*. (XLSX 336 kb)

Additional file 2: **Figure S1.** Phylogenetic classification of pineapple PKs. The phylogenetic tree was constructed with amino sequences of the kinase domain using FastTree 2.1.9 with maximum-likelihood method. Families were highlighted with different colors. (PDF 286 kb)

Additional file 3: **Figure S2.** Chromosomal locations of pineapple PK genes. **Figure S3.** Subcellular localizations of pineapple PK genes in seven large groups predicted by CELLO. **Figure S4.** GO analysis of the 95 tandemly duplicated PK genes in pineapple. The size of each slice in the pie chart indicates the relative abundance of that GO term in the pineapple kinome. **Figure S5.** Heatmaps of the expression profiles of pineapple PK families in 14 different tissues with hierarchical clustering. The heatmaps were generated using R. **Figure S6.** Coexpression networks of pineapple PK families in 14 different tissues. Nodes indicated families and edges indicated significant coexpression between families. All of Pearson correlation coefficients of coexpression events were significant at the 0.01 significance level (*p*-value). **Figure S7.** Coexpression network analysis of PK genes in cluster 1 in green leaf tip and white leaf base during 24 h period. Nodes indicated genes, and edges indicated significant coexpression between genes. All of Pearson correlation coefficients of coexpression events were significant at the 0.01 significance level (*p*-value). **Figure S8.** Coexpression network analysis of PK genes which were generated by segmental (A) and tandem (B) duplication in green leaf tip and white leaf base during 24 h period. Nodes indicated genes, and edges indicated significant coexpression between genes. The absolute value of Pearson correlation coefficients > 0.9, and *p* < 0.01. Different line colors indicate either positive (blue) or negative (red) correlations. (DOCX 3152 kb)

## Abbreviations

AGC: PKA-PKG-PKC
AS: Alternative splicing
CAM: Crassulacean acid metabolism
CAMK: Calcium- and calmodulin-regulated kinase
CK1: Casein kinase 1
CMGC: Cyclin-dependent kinases, mitogen-activated protein kinases, glycogen synthase kinase and cyclin-dependent-like kinases
FPKM: Fragments per kilobase of exon model per million fragments mapped
GO: Gene ontology
HMM: Hidden Markov model
*Ka*: non-synonymous substitution per site
*Ks*: Synonymous substitution per site
LRR: Leucine-rich repeat
ML: Maximum-likelihood
PCC: Pearson correlation coefficient
PK: Protein kinase
RLCK: Receptor-like cytoplasmic kinase
RLK: Receptor-like kinase
RNA-Seq: RNA sequencing
STE: Sterile kinase
TK: Tyrosine kinase
TKL: Tyrosine kinase-like kinase
