# Supplementary figures and images for: The kinome of pineapple: catalog and insights into functions in crassulacean acid metabolism plants
Source: BMC Plant Biol. 2018 Sep 18;18:199. doi: 10.1186/s12870-018-1389-z (PMC6145126; doi:10.1186/s12870-018-1389-z)

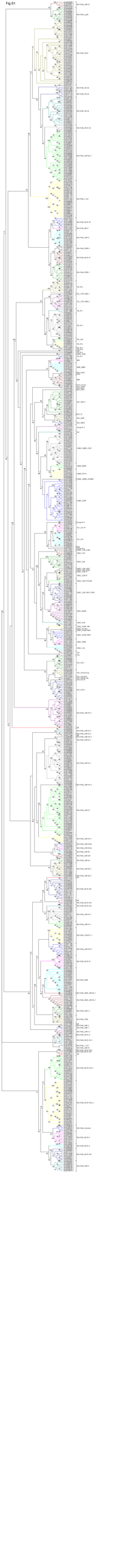

Supplement: Supplementary file 2 — Figure S1. Phylogenetic classification of pineapple PKs. The phylogenetic tree was constructed with amino sequences of the kinase domain using FastTree 2.1.9 with maximum-likelihood method. Families were highlighted with different colors. (PDF 286 kb) [file 12870_2018_1389_MOESM2_ESM.pdf]
