# Supplementary material for: The kinome of pineapple: catalog and insights into functions in crassulacean acid metabolism plants
Source: BMC Plant Biol. 2018 Sep 18;18:199. doi: 10.1186/s12870-018-1389-z (PMC6145126; doi:10.1186/s12870-018-1389-z)
Supplement: Supplementary file 3 — Figure S2. Chromosomal locations of pineapple PK genes. Figure S3. Subcellular localizations of pineapple PK genes in seven large groups predicted by CELLO. Figure S4. GO analysis of the 95 tandemly duplicated PK genes in pineapple. The size of each slice in the pie chart indicates the relative abundance of that GO term in the pineapple kinome. Figure S5. Heatmaps of the expression profiles of pineapple PK families in 14 different tissues with hierarchical clustering. The heatmaps were generated using R. Figure S6. Coexpression networks of pineapple PK families in 14 different tissues. Nodes indicated families and edges indicated significant coexpression between families. All of Pearson correlation coefficients of coexpression events were significant at the 0.01 significance level (p-value). Figure S7. Coexpression network analysis of PK genes in cluster 1 in green leaf tip and white leaf base during 24 h period. Nodes indicated genes, and edges indicated significant coexpression between genes. All of Pearson correlation coefficients of coexpression events were significant at the 0.01 significance level (p-value). Figure S8. Coexpression network analysis of PK genes which were generated by segmental (A) and tandem (B) duplication in green leaf tip and white leaf base during 24 h period. Nodes indicated genes, and edges indicated significant coexpression between genes. The absolute value of Pearson correlation coefficients > 0.9, and p < 0.01. Different line colors indicate either positive (blue) or negative (red) correlations. (DOCX 3152 kb) [file 12870_2018_1389_MOESM3_ESM.docx]

**Figure S2**

**Figure S2** Chromosomal locations of pineapple PK genes**.**

**

Figure S3**

**Figure S3** Subcellular localizations of pineapple PK genes in seven large groups predicted by CELLO.

**Figure S4**

**Figure S4** GO analysis of the 95 tandemly duplicated PK genes in pineapple. The size of each slice in the pie chart indicates the relative abundance of that GO term in the pineapple kinome.

**Figure S5**

**Figure S5.** Heatmaps of the expression profiles of pineapple PK families in 14 different tissues with hierarchical clustering. The heatmaps were generated using R.

**Figure S6**

**Figure S6** Coexpression networks of pineapple PK families in 14 different tissues. Nodes indicated families and edges indicated significant coexpression between families. All of Pearson correlation coefficients of coexpression events were significant at the 0.01 significance level (*p*-value).

**Figure S7**

**Figure S7** Coexpression network analysis of PK genes in cluster 1 in green leaf tip and white leaf base during 24 h period. Nodes indicated genes, and edges indicated significant coexpression between genes. All of Pearson correlation coefficients of coexpression events were significant at the 0.01 significance level (*p*-value).

**Figure S8**

**Figure S8** Coexpression network analysis of PK genes which were generated by segmental (A) and tandem (B) duplication in green leaf tip and white leaf base during 24 h period. Nodes indicated genes, and edges indicated significant coexpression between genes. The absolute value of Pearson correlation coefficients > 0.9, and *p* < 0.01. Different line colors indicate either positive (blue) or negative (red) correlations.
